# Model Test Study on Deformation of Snowflake Shaped Steel Sheet Pile Based on OFDR

**DOI:** 10.3390/s21217062

**Published:** 2021-10-25

**Authors:** Lei Gao, Zhongquan Xu, Quan Wang, Zhenlei Zhang, Ping Li

**Affiliations:** 1 Key Laboratory of Ministry of Education for Geomechanics and Embankment Engineering, Hohai University, Nanjing 210098, China; 18262622179@163.com (Z.X.); wq1879056258@163.com (Q.W.); 18268086843@163.com (Z.Z.); lipings0110@163.com (P.L.); 2 Research Institute of Geotechnical Engineering, Hohai University, Nanjing 210098, China

**Keywords:** snow shaped steel sheet pile, OFDR, optical fiber, pile strain, model test

## Abstract

As a newly developed pile foundation, the snowflake shaped steel sheet pile is composed of three Y-shaped sections with an included angle of 120° and has a large specific surface area, which can give full play to the side friction of pile and improve the bearing capacity of single pile. At the same time, the snowflake shaped steel sheet pile has a high strength, relatively few materials, and it has good prospects with engineering applications. In order to accurately grasp the mechanical characteristics of the snowflake shaped steel sheet pile, this paper carried out the model test of snowflake shaped steel sheet pile based on OFDR (optical frequency domain reflector) distributed optical fiber sensor technology. The results show that: (1) OFDR distributed optical fiber sensing technology can effectively monitor the strain of snowflake steel sheet pile; (2) under the vertical load, the strain of snowflake steel sheet pile decreases along the length of the pile; (3) the strain of the same section of snowflake steel sheet pile is different at different positions, the strain at the junction between web and web is basically the same as the junction between web and flange, and the strain of the pile shaft on the flange edge is larger.

## 1. Introduction

Pile is the most common form of foundation reinforcement, the section forms of which are various, such as square, circular, triangular, and so on. In recent years, with the increasing requirements of energy conservation and emission reduction and green environmental protection in the field of infrastructure construction, some new special-shaped piles have been gradually applied [1,2,3,4,5,6,7,8]. Under the same bearing capacity, special-shaped piles can reduce the use of materials and have good economic benefits.

**Figure 1:**
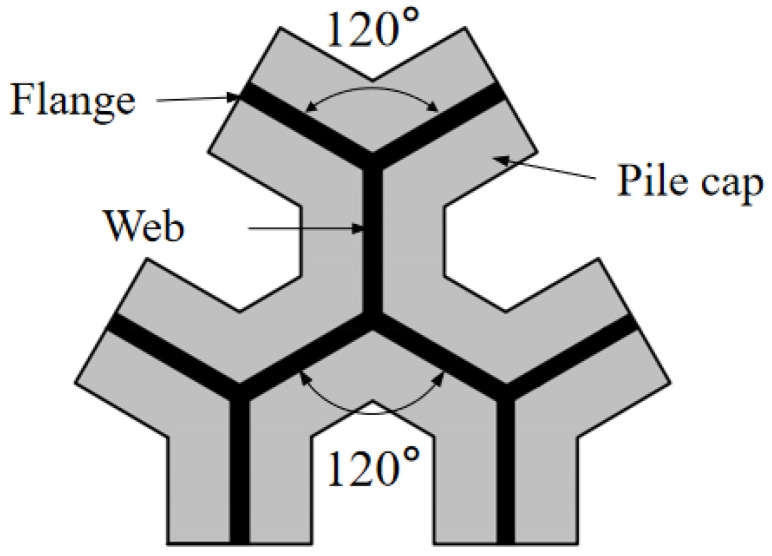
Structural diagram of snowflake shaped steel sheet pile.

The snowflake shaped steel sheet pile [9] technology which was developed by Hohai University is a foundation treatment patented technology with independent intellectual property rights (Patent No: ZL201810192602.X). The structural diagram of the snowflake shaped steel sheet pile is shown in Figure 1. The cross section of the pile shaft is composed of three Y-shaped sections with an included angle of 120°. The star shaped design with axial symmetry increases the overall stiffness of the steel sheet pile, which can effectively control the pile deformation. The snowflake shaped steel sheet pile has a large specific surface area, which can fully play the role of pile side friction; the steel has high strength, which can drive into hard soil without damage to the pile shaft. The steel sheet pile is thin and it has little impact on the adjacent buildings. The snowflake shaped steel sheet pile can save materials, it can be welded on site and have large reinforcement area, and it is convenient for transportation and production. This new pile technology is suitable for solving the problems of bump at bridge head, uneven settlement, soft soil foundation reinforcement and emergency construction.

In recent years, the distributed optical fiber monitoring technologies are used for engineering monitoring [10], such as BOTDR [11,12], BOTDA [13], BOFDA [14], OFDR [15], and so on. The comparison of various distributed optical fiber sensing technologies is shown in Table 1. In terms of measurement accuracy and spatial resolution, OFDR (optical frequency domain reflector) technology is the best one among the distributed optical fiber monitoring technologies, and it has been used in the field of geotechnical engineering monitoring [16,17,18]. Liu et al. [19] applied OFDR optical fiber sensing technology to a cross rock pillar excavation model test pile to monitor the horizontal strain of the model. Gao et al. [20] carried out a small model test to study the deformation characteristics of PCC pile using OFDR technology.

Due to the fact that the snowflake shaped steel sheet pile is currently in the stage of technical research and development, the deformation of this pile is still not clear, and there are no effective ways to obtain the strain information of the pile. In order to obtain the deformation of snowflake shaped steel sheet pile, this paper carried out a single snowflake steel sheet pile model test. The optical fiber sensors and strain gauges were used to monitor the deformation of snowflake steel sheet pile under vertical load at the same time.

## 2. Principle of OFDR Technology

**Figure 2:**
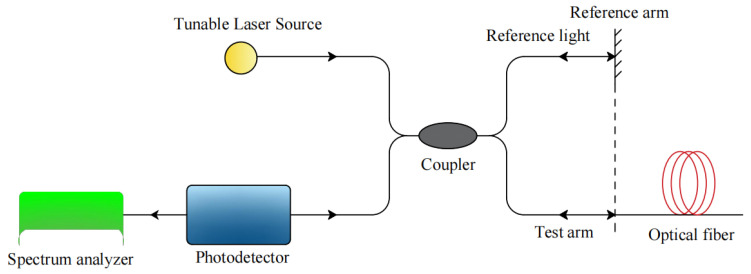
Principle of OFDR technology [20].

The OFDR technology is a strain measurement technology based on Rayleigh scattering light proposed by Eickoff in 1981 [21]. Its basic principle is to use continuous wave frequency scanning technology, heterodyne interferometry, periodic linear wavelength scanning light source and coupler to connect to the reference arm and signal arm, respectively. The local oscillator of the reference arm and the Rayleigh backscattering signal of the signal arm have different optical paths, so they carry different frequencies. Therefore, beat frequency interference occurs between them, and the beat frequency of the interference signal is proportional to the distance of the backscattering position of the signal arm. After fast Fourier transform, the information of the fiber Rayleigh backscattering signal in the range domain can be obtained. The working principle of OFDR is shown in Figure 2.

Rayleigh scattering in optical fiber is caused by the random change of the refractive index of the optical fiber material. Because the random distribution is relatively stable, the optical fiber can be regarded as a kind of weak fiber Bragg grating with random period everywhere. When the fiber strain changes, the spectrum of backscattered signal will shift in frequency, The magnitude is proportional to the strain produced by the fiber. The optical fiber strain can be calculated by Equation (1). The distributed strain information of the whole fiber can be obtained by scanning all parts of the fiber with the moving window.

Δ*ν* = *C*_*ε*_·Δ*ε* (1)

In the equation, Δ*ν* is the value of frequency drift of Rayleigh spectrum, Δ*ε* is the change of strain of optical fiber relative to the initial value, and *C_ε_* is the strain proportional coefficient of optical fiber.

## 3. Model Test of Pile Deformation

**Figure 3:**
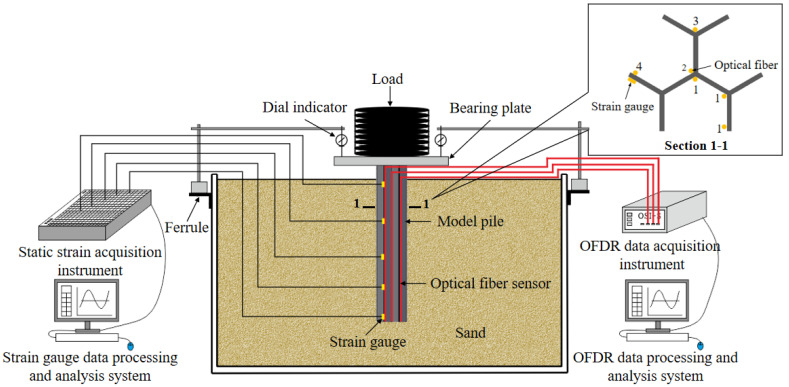
Schematic diagram of test design.

The test is carried out in a plexiglass model tank with an inner wall size of 60 cm × 40 cm × 40 cm, and the overall design diagram of the model test is shown in Figure 3.

### 3.1. Test Soil Sample

In order to do the test, it was decided to select fine sand as the soil around the pile. Its main physical parameters are shown in Table 2.

### 3.2. Model Pile

**Figure 4:**
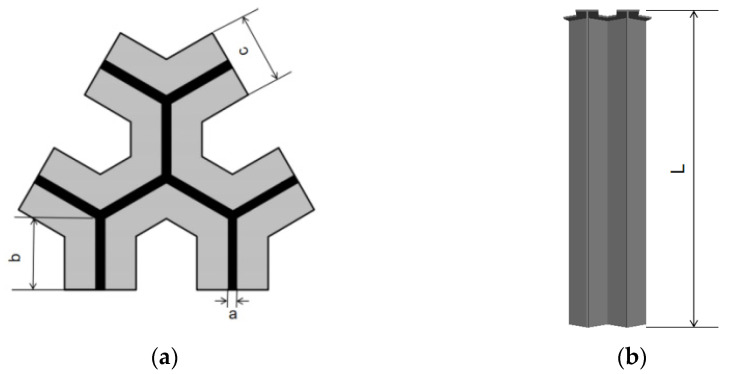
Dimension diagram of snowflake shaped steel sheet pile model pile. (**a**) Section; (**b**) Elevation.

The steel of model pile is quasi steel grade C45E4, the design height of model pile is 35 cm, and the thickness of pile shaft and pile cap is 2.5 cm. In order to ensure the integrity of the pile, the wire cutting technology is used to make the pile. The section size of model pile is shown in Table 3, and the schematic diagram is shown in Figure 4.

### 3.3. Data Acquisition Equipment

The data acquisition equipment used in this test include OSI-S distributed optical fiber sensor and static strain acquisition instrument. The principle of OSI-S distributed optical fiber sensor is based on OFDR technology. It has the advantages of high spatial resolution, large dynamic range and high test sensitivity. It can carry out distributed measurement of temperature, strain and other parameters. The spatial resolution can reach 1 mm within 50 m sensing range and 1 cm within 100 m sensing range. The device can measure thousands of sensing points on one optical fiber at the same time, and is used in the field of short-distance, high-resolution and high-precision sensing. The technical parameters of OSI-S distributed optical fiber sensor are shown in Table 4.

### 3.4. Sensor Layout

The optical fiber used in this test is φ 900 single-mode fiber, which is widely used in civil engineering deformation monitoring and experimental research. The optical fiber itself is thin and fragile, which is easy to be damaged and broken. Therefore, the protective measures for the sensing optical fiber shall be taken during the deployment of optical fiber sensor. Considering that the steel sheet pile is thin, and slotting will affect its integrity, the optical fiber is directly arranged on the surface of the steel sheet pile. Firstly, we paste a piece of double-sided adhesive tape at a certain distance at the position where the optical fiber needs to be laid on the steel sheet pile, fix the optical fiber sensor on the surface of the steel sheet pile at a fixed point, and then fix it along the sensing optical fiber with epoxy resin to realize full coverage packaging protection. In this process, it will ensure that the optical fiber is closely attached to the pile body, and the optical fiber and the model pile can be deformed in coordination.

**Figure 5:**
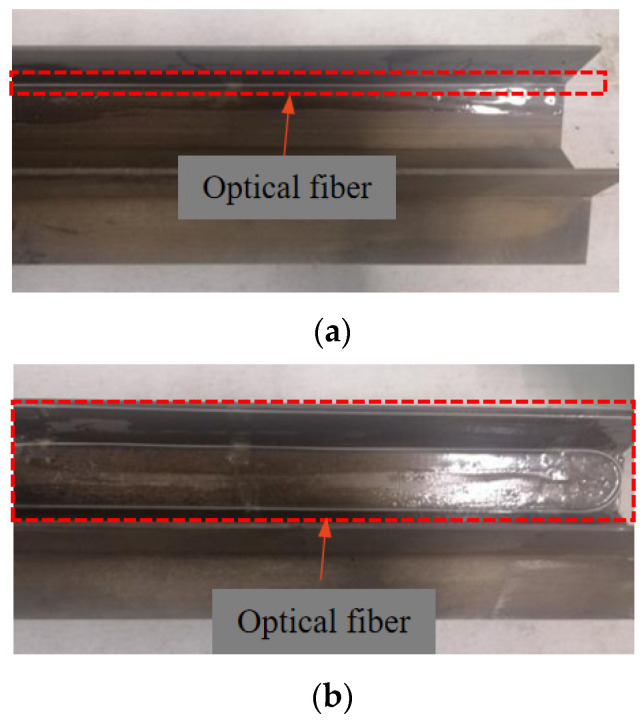
Sensor layout diagram: (**a**) 1-shaped layout; (**b**) S-shaped layout.

The layout diagram of sensors in this test is shown in section 1-1 in Figure 3. Four optical fibers and a group of strain gauges are arranged. Among them, No. 1 optical fiber is arranged in an S-shape to connect the three positions of the junction between web and web, the junction between web and flange, and the flange edge. No. 2, 3 and 4 optical fibers are respectively arranged in 1-shape at these three locations. The two layout forms of optical fiber on the model pile are shown in Figure 5a,b. As the control group of optical fiber 4, strain gauges are arranged at the corresponding position of optical fiber 4. A total of 5 strain gauges are arranged with a spacing of 8 cm, and the distance between the first strain gauge and the pile top is 2 cm. Since the test is conducted in the indoor model tank and the rapid loading method is adopted, the temperature hardly changes, so there is no need for temperature compensation in this test.

### 3.5. Sand Landfill and Data Collection

**Figure 6:**
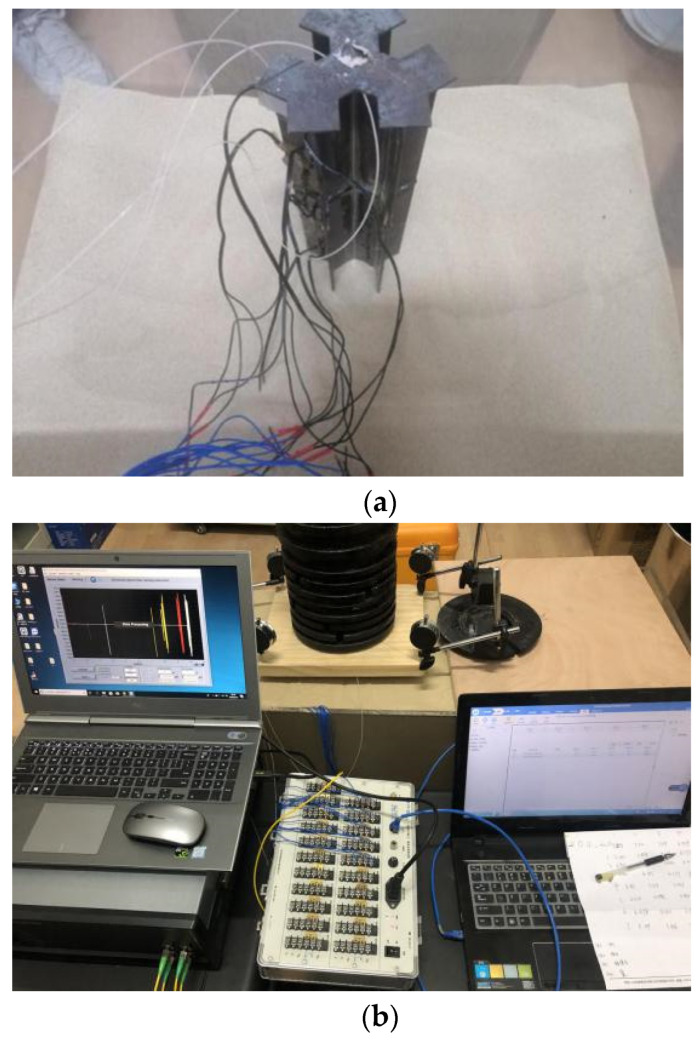
Sand landfill and data collection. (**a**) Placing model piles vertically; (**b**) loading and data acquisition.

In the process of landfill, the snowflake shaped steel sheet pile should be vertical to avoid excessive inclination and affect the test results. Considering the size difference between the model pile cap and the weight, a bearing plate (20 cm × 20 cm) is fixed on the top of the pile to ensure the stability of the loading. The dial indicators are installed at the four corners of the bearing plate to measure the settlement of the pile top. The four optical fiber sensors are inserted into the four channels of OSI-S distributed optical fiber acquisition instrument, respectively, the strain gauges are connected into the static strain acquisition instrument. The process of sand landfill and data collection are shown in Figure 6.

## 4. Analysis of Test Results

### 4.1. Load-Settlement Curve

**Figure 7:**
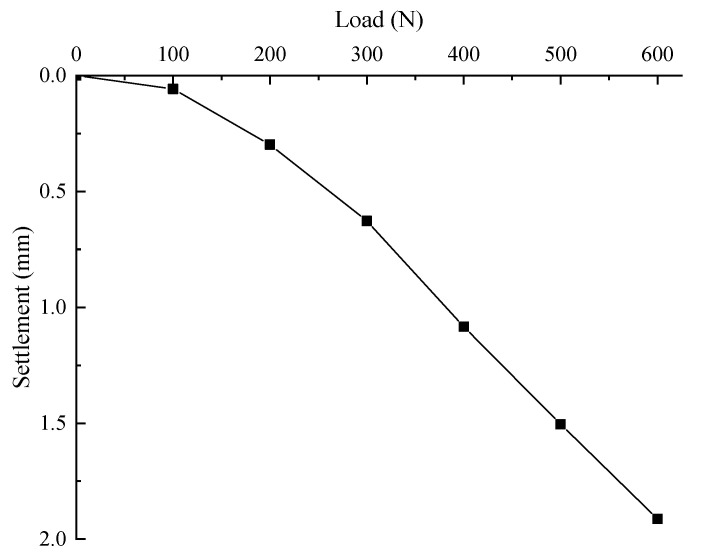
Load-settlement curve.

Figure 7 shows the load settlement curve of pile top. It can be seen from the figure that under the applied load, the curve is of slow deformation and there is no steep drop section. Before 300 N, the slope of the curve increases continuously. After 300 N, the load settlement curve tends to be a straight line, the settlement increment of the pile top under various loads is basically unchanged. The maximum settlement of pile top is 1.913 mm.

### 4.2. Comparison of Monitoring Data between Distributed Optical Fiber and Strain Gauge

**Figure 8:**
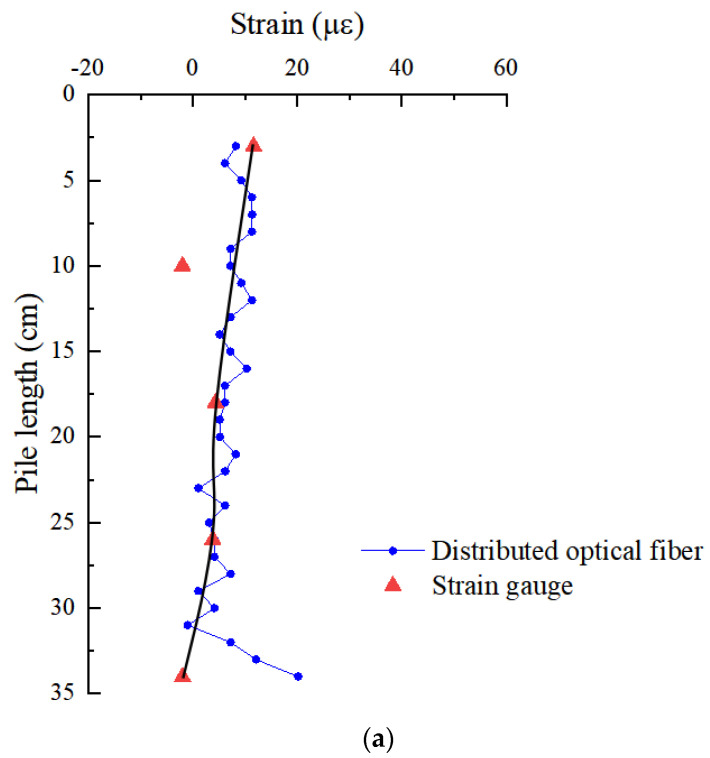

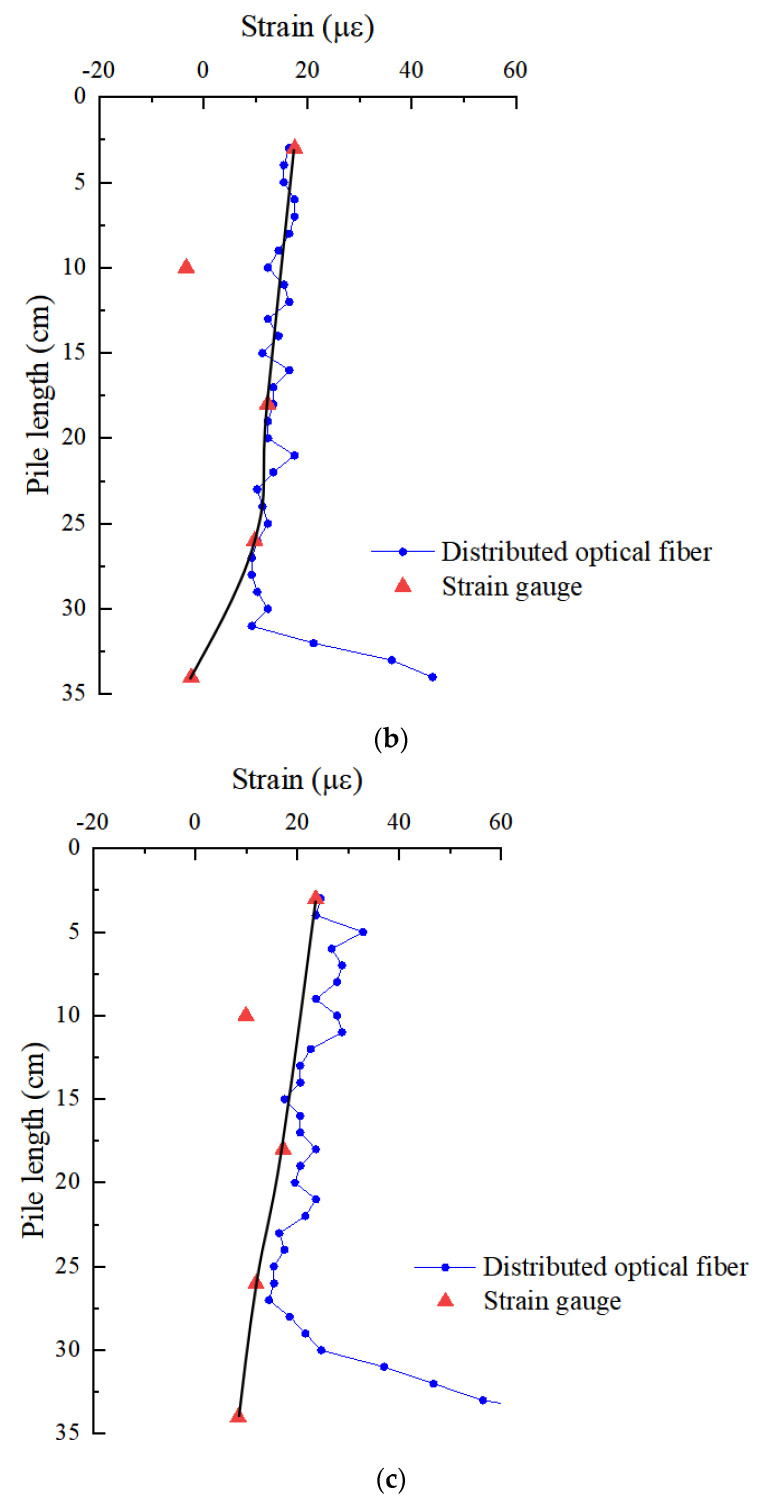
Comparison of monitoring results of distributed optical fiber and strain gauge under different loads: (**a**) 200 N; (**b**) 400 N; (**c**) 600 N.

In order to verify the effectiveness of OFDR distributed optical fiber monitoring technology in the deformation monitoring of snowflake shaped steel sheet pile, the distributed optical fiber and strain gauge are used to monitor the deformation of snowflake steel sheet pile at the same time, and the data measured by the two are compared and analyzed. Figure 8 is a comparative analysis of the strain of pile shaft under different loads of No. 4 optical fiber and strain gauge arranged on the same flange edge.

It can be seen from the figure that the strain values monitored by strain gauge are near the strain curve monitored by distributed optical fiber. Except that the monitoring results of the second and fifth strain gauges are quite different from the results of distributed optical fiber monitoring, the strain measured by the other three strain gauges and distributed optical fiber are close to each other; the maximum error between the two is less than 27%. The variation law of the two is basically the same, which is decreasing along the depth direction. In the effective data section, the monitoring results of distributed optical fiber and strain gauge are basically consistent, which shows that the distributed optical fiber can effectively monitor the strain of snowflake shaped steel sheet pile. Moreover, the advantage of distributed optical fiber in monitoring the deformation details of pile foundation is better than that of strain gauge, which can clearly know the strain change at the depth of pile body under each load level. This provides more reliable basis for the design.

### 4.3. Raw Data Analysis of Distributed Optical Fiber

**Figure 9:**
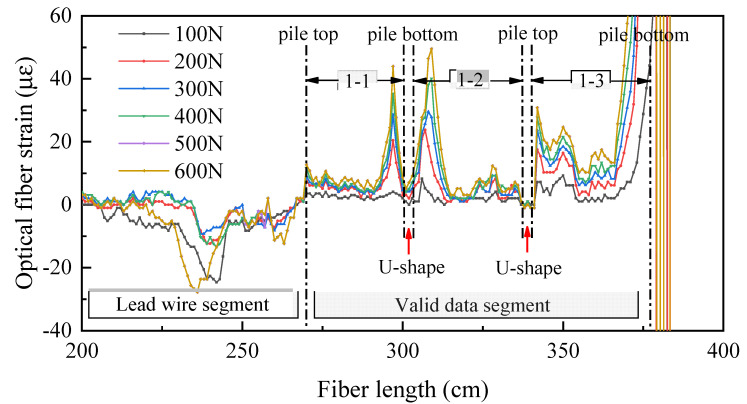
The data of No. 1 optical fiber: 1-1 is strain of pile shaft at the junction between web and web; 1-2 is strain of pile shaft at the junction between web and flange; 1-3 is strain of pile shaft on flange edge.

**Figure 10:**
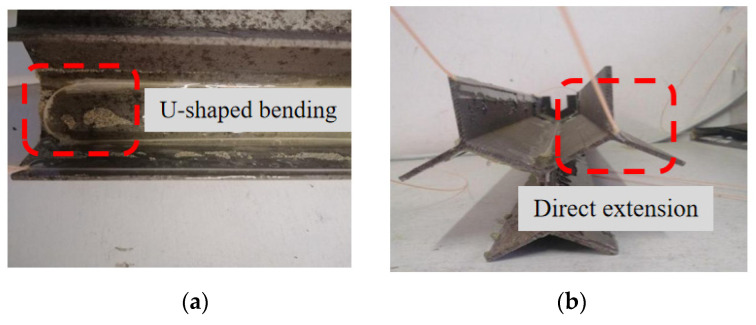
Layout of optical fiber at pile bottom. (**a**) U-shaped bending; (**b**) direct extension.

Since the optical fiber on the pile is only a part of the whole optical fiber, it is necessary to judge and extract the monitoring data belonging to the pile. Taking the data of No. 1 optical fiber as an example, the original data of optical fiber are analyzed. As shown in Figure 9, the data corresponding to the pile shaft is divided into three sections. As the No. 1 optical fiber is S-shaped, there are two forms of optical fiber at the pile bottom, one is U-shaped bending, the other is directly inserted into the soil (as shown in Figure 10). Under the action of load, the pile will produce settlement. Because the optical fiber is directly laid on the surface of the model pile, the optical fiber near the pile bottom will be subject to the upward external force of sand during the settlement process, which will have a certain impact on the fiber-optic pile body strain measurement.

It is found that the strain near the pile bottom will first increase and then decrease with the optical fiber U-shaped bending at the pile bottom. When the optical fiber is directly inserted into the soil, the strain near the pile bottom will increase rapidly. These two distributions are inconsistent with the actual stress of conventional piles.

### 4.4. Verification Test

In order to further confirm the phenomenon of the strain near the pile bottom at the junction between web and web, the junction between web and flange first increases and then decreases with the optical fiber U-shaped bending. The phenomenon of a sharp increase near the pile bottom when optical fiber is directly inserted into the soil is caused by the test error; they are not the actual deformation of the pile, a verification test is conducted by placing the model pile directly on the ground without sand filling.

**Figure 11:**
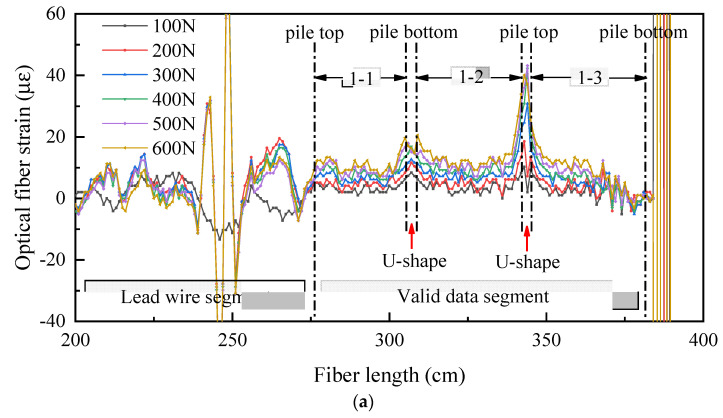

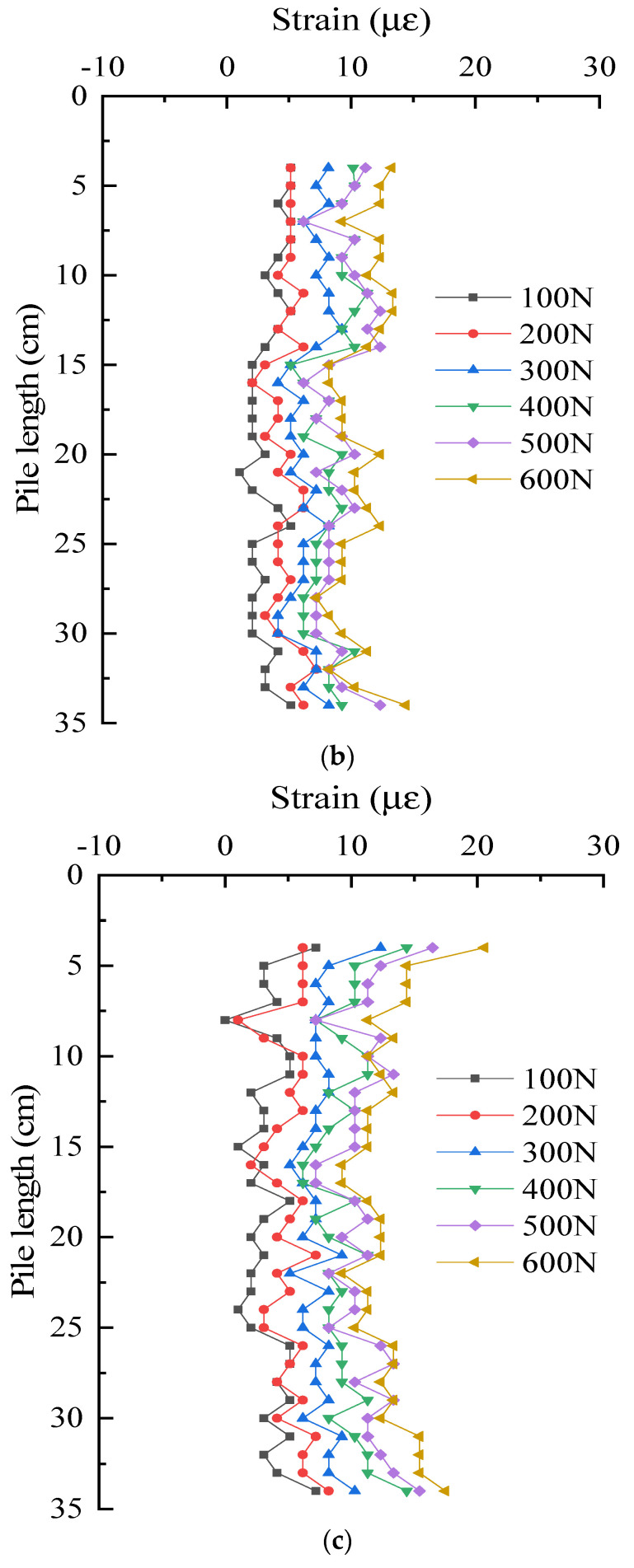

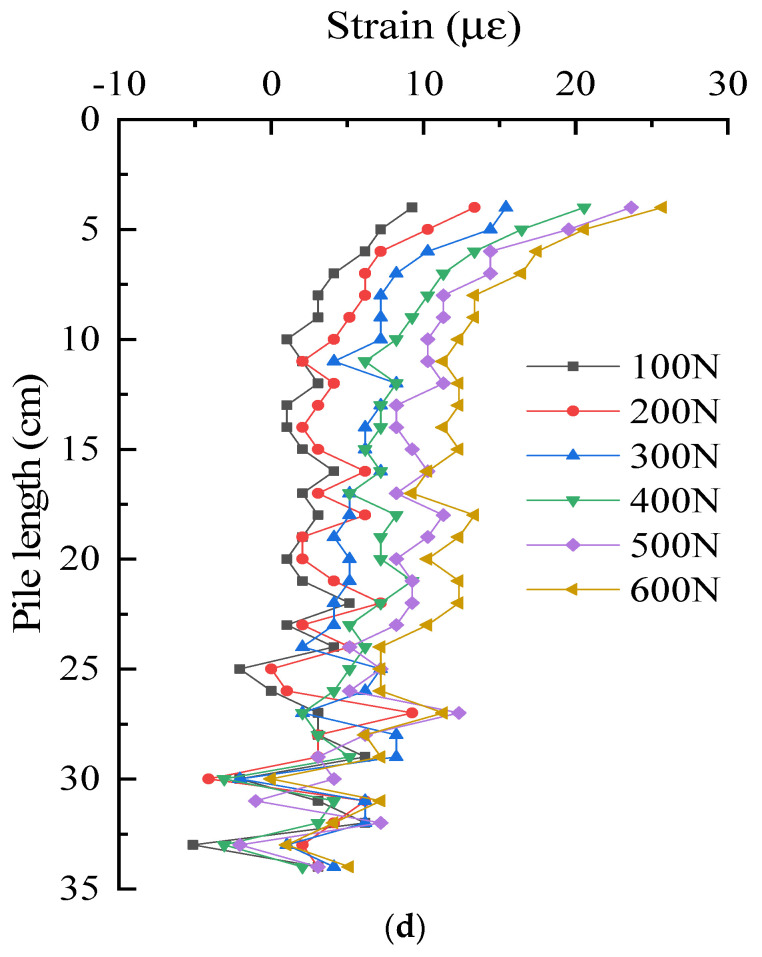
Test results without sand filling. (**a**) The raw data of optical fiber without sand filling; (**b**) 1-1: strain of pile shaft at the junction between web and web; (**c**) 1-2: strain of pile shaft at the junction between web and flange; (**d**):1-3: strain of pile shaft on flange edge.

Figure 11 shows the strain distribution of pile shaft at different positions without sand filling. It can be seen from the figure regarding the strain of pile shaft at three locations, neither increase first and then decrease nor increase sharply near the pile bottom, it is relatively gentle as a whole. There is a slight decrease trend from the pile top to the pile bottom. The results of the verification test show that the distribution of the strain near the pile bottom at the junction between web and web and web and flange first increases and then decreases; the sharp increase of the strain near the pile bottom on the flange edge is caused by the test error, they are not the actual deformation of the snowflake shaped steel sheet pile.

### 4.5. Strain Distribution of Pile Shaft

**Figure 12:**
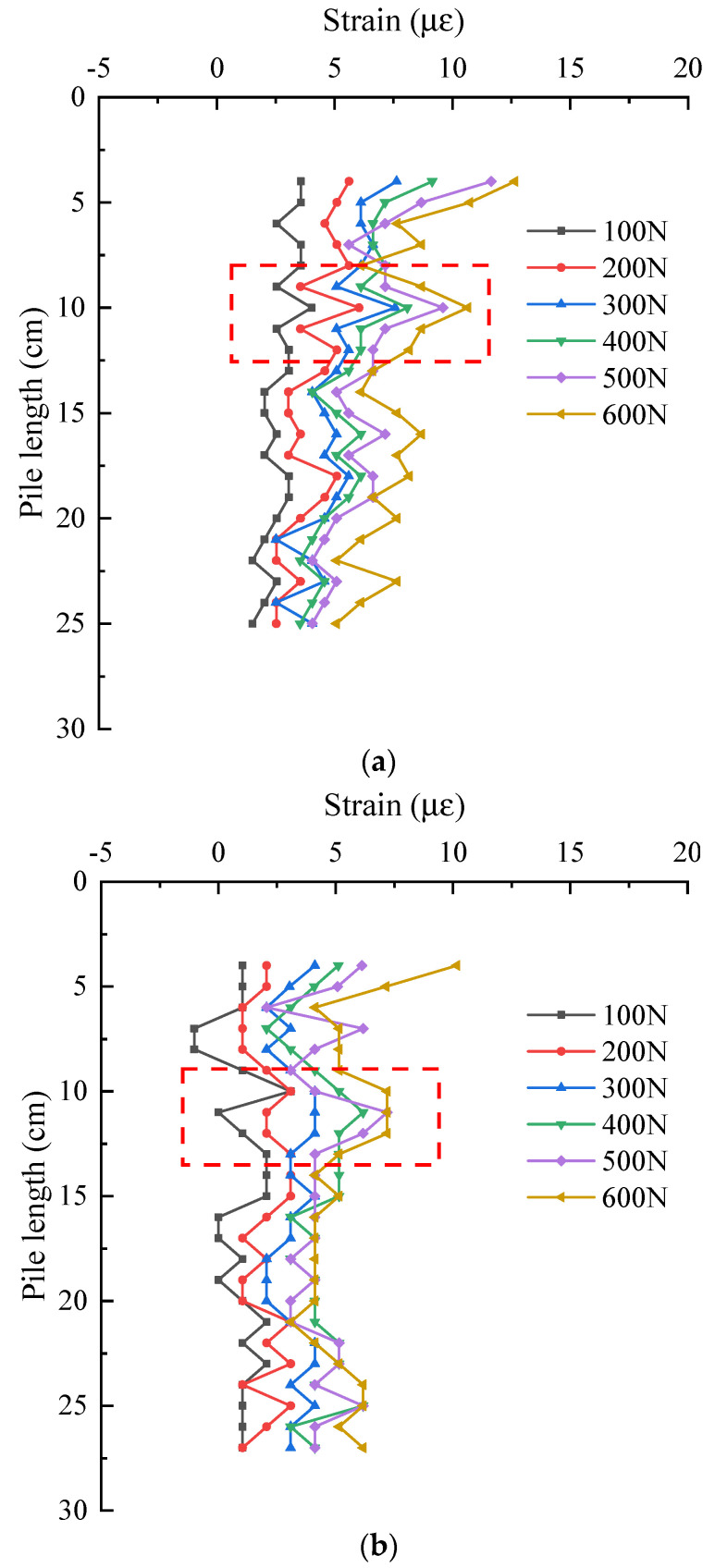
Strain distribution of pile shaft at the junction of web and web. (**a**) S-shaped layout—No. 1 optical fiber; (**b**) 1-shaped layout—No. 2 optical fiber.

**Figure 13:**
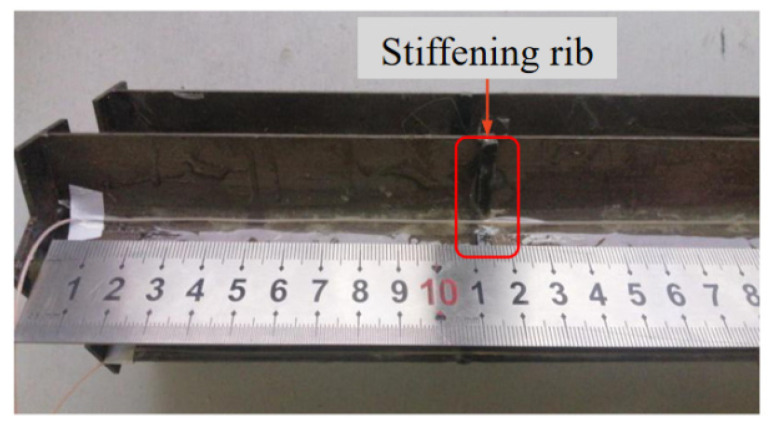
The fiber is jacked up at the stiffener rib.

The following is a detailed analysis of the strain distribution characteristics of the effective section of the pile at different positions. Figure 12 shows the strain distribution of pile shaft at the junction between web and web under two groups of different layout methods. They are the data of No. 1 optical fiber web and web junction and No. 2 optical fiber. It can be seen from the figure that with the increase of the pile top load, the strain at the same position of the pile shaft is increasing. Under the same load, in S-shape layout, the strain of pile shaft decreases in the range of 0~25 cm. In 1-shape layout, the strain of pile shaft decreases in the range of 0~27 cm. The strain variation law of the two groups in their respective reduced sections is basically the same, and the strain values are also very close. As shown in the data in the red rectangle in Figure 12a,b, there is a slight increase in both data about 11 cm away from the pile top. The reason is as shown in Figure 13, the model pile is welded with a stiffener 11 cm away from the pile top, resulting in the optical fiber 11 cm away from the pile top being jacked up by the stiffener. At this time, the optical fiber in this section does not fit closely with the pile body, and the stress of the optical fiber in this section deviates from the actual stress of the pile.

**Figure 14:**
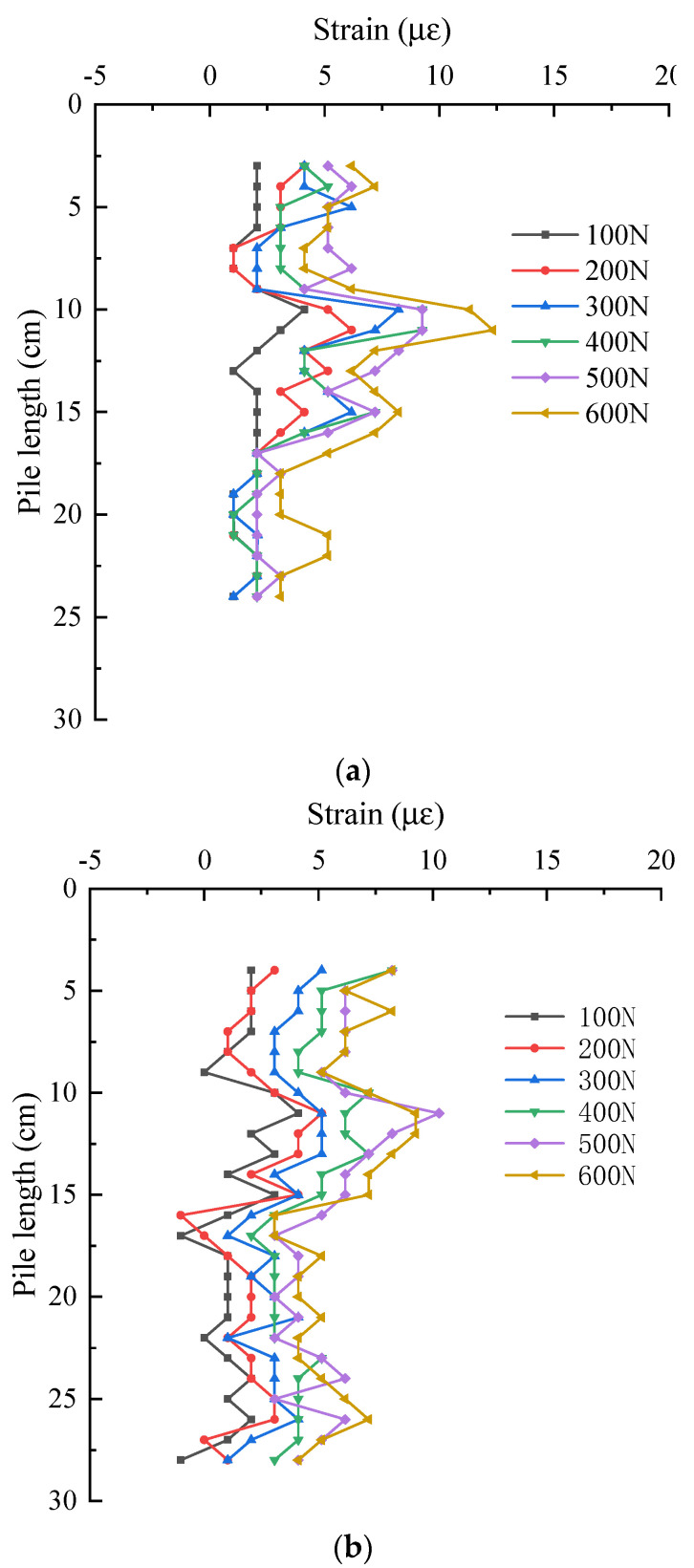
Strain distribution of pile shaft at the junction of web and flange. (**a**) S-shaped layout—No. 1 optical fiber; (**b**) 1-shaped layout—No. 3 optical fiber.

Figure 14 shows the strain distribution of pile shaft at the junction between web and flange under two groups of different layout methods, they are the data of No. 1 optical fiber web and flange junction and No. 3 optical fiber. It can be seen from the figure that the strain distribution of pile shaft is roughly the same as that at the junction between web and web.

**Figure 15:**
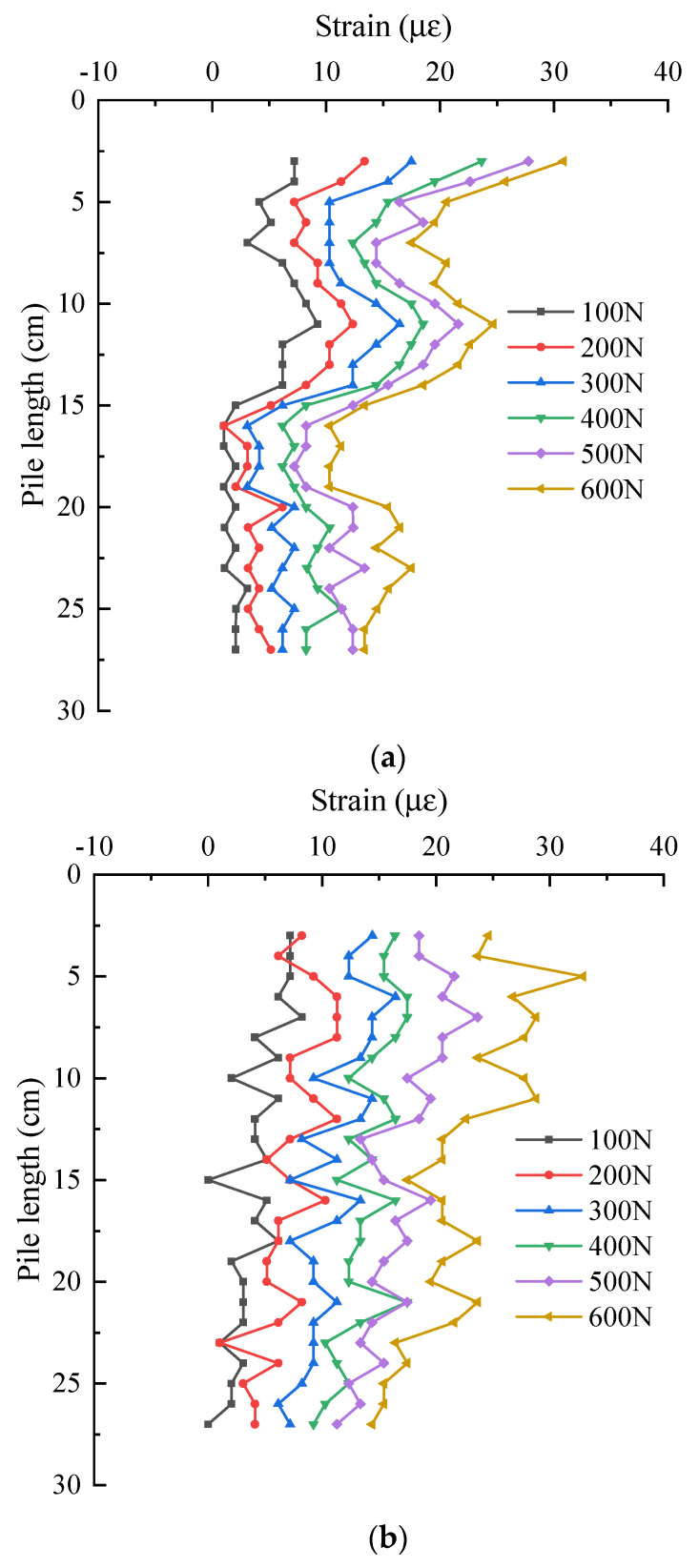
Strain distribution of pile shaft on the flange edge. (**a**) S-shaped layout—No. 1 optical fiber; (**b**) 1-shaped layout—No. 4 optical fiber.

Figure 15 shows the strain distribution of pile shaft on the flange edge of two groups of different layout methods, they are the data of No. 1 optical fiber flange edge and No. 4 optical fiber. It can be seen from the figure that with the increase of the pile top load, the strain at the same position of the pile is increasing. Under the same load, the strain distribution along the length of the pile is basically the same, except for the influence range of the stiffener, and they all decrease along the length of pile.

**Figure 16:**
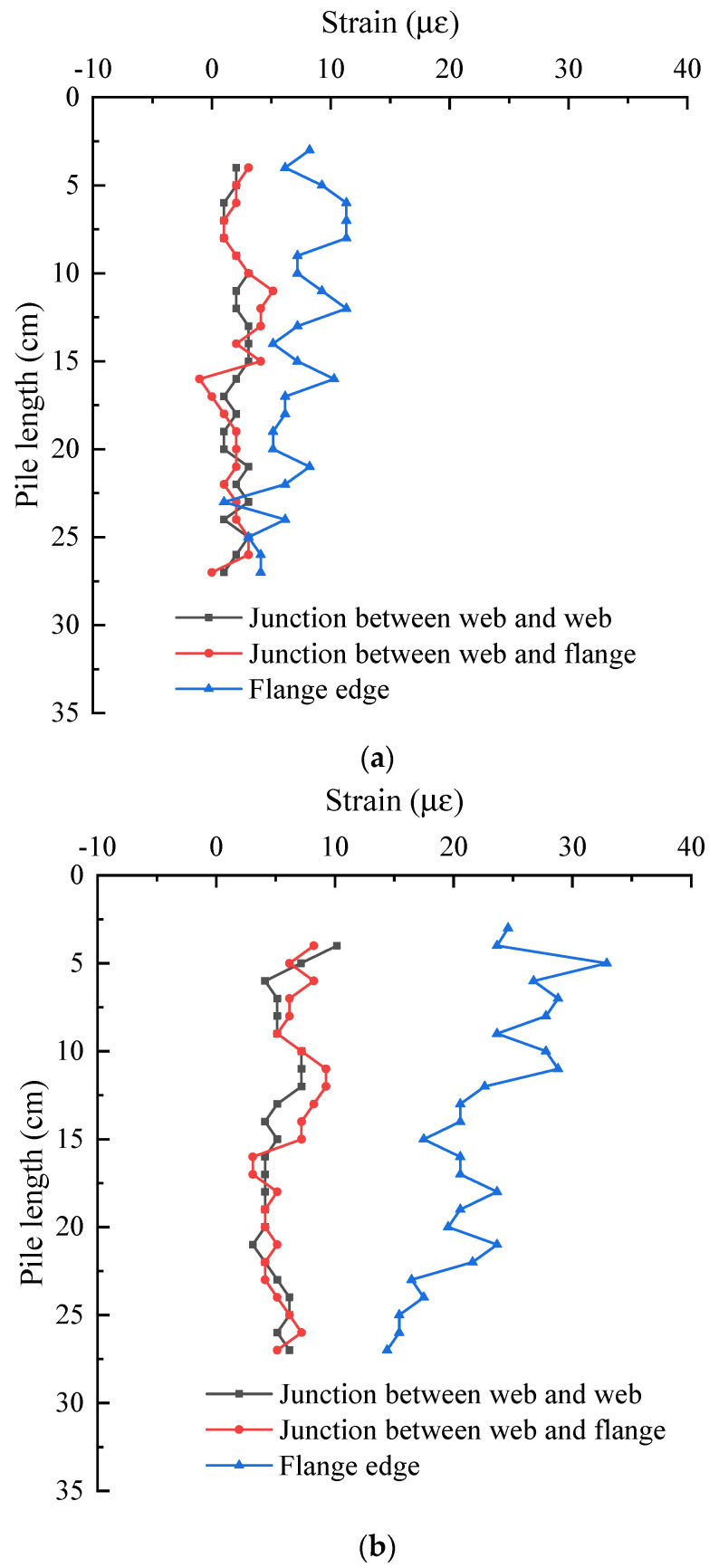
Strain comparison of piles at different positions under the same load: (**a**) 200 N; (**b**) 600 N.

By comparing the above three groups of curves, it is found that the strain of snowflake shaped steel sheet pile decreases from top to bottom along the pile length. In order to compare and analyze the strain difference of pile shaft at different positions, the strain of three positions of pile shaft under 200 N and 600 N is compared, as shown in Figure 16. It can be seen that the strain distribution of pile shaft at three locations under 200 N and 600 N are basically the same, the strain of the pile shaft at the junction between web and web, between web and flange are basically the same, and the maximum error of the two in the whole pile length direction is no more than 40%. However, the strain of the pile on the flange edge is larger. The strain on the flange edge is the largest and increases more and more with the increase of load. Compared with the junction between web and web and the junction between web and flange, the strain on the flange edge increases by about 4 με on average under 200 N load and increases by about 16.4 με under 600 N load. The reason for this difference is that the junction between web and web and the junction between web and flange are the intersection of three steel plates, which have high stiffness, and are not easy to deform. There is only one steel plate on the flange, the stiffness is not greater than that at the junction, and the deformation is larger.

## 5. Conclusions

In this paper, the model test of snowflake shaped steel sheet pile is described in detail. The strain data of pile collected by distributed optical fiber and strain gauge in the test are studied and analyzed. The following conclusions are obtained:

1. Considering the results of OFDR distributed and strain gauge monitoring technology, it can be concluded that the strain of snowflake shaped steel sheet pile decreases along the pile length under the vertical load.
2. The strain of the pile shaft at different positions of the same section of the snowflake steel sheet pile is different. The strain of the pile shaft at the junction between web and web and between web and flange are basically the same, but the strain of the pile on the flange edge is larger.
3. OFDR distributed sensing technology can effectively monitor the strain of snowflake shaped steel sheet pile. Compared with the data measured by strain gauge, it is found that the two monitoring results are similar, and OFDR distributed sensing technology can monitor more information which shows the advantages of distributed optical fiber in monitoring the deformation details of pile. It can clearly identify the strain distribution of pile under load, and therefore will provide more reliable basis for design.

## Figures and Tables

**Table 1:** Comparison of various distributed optical fiber sensing technologies.

Technical Name	Strain Accuracy /(10^−^^6^)	Spatial Resolution /(m)	Test Length /(km)
BOTDR	±20	1.000	80.0
BOTDA	±2	0.050	20.0
BOFDA	±2	0.200	50.0
OFDR	±1	0.001	0.1

**Table 2:** Mechanical parameters of soil material.

Soil Name	Density *ρ*/(kg/m^3^)	Elastic Modulus *E*/(MPa)	Poisson’s Ratio	Cohesion *C*/(kPa)	Internal Friction Angle *φ*/(°)
Fine sand	1600	8	0.3	0	30

**Table 3:** Parameters of model pile shaft.

Name	Unit	Snowflake Shaped Steel Sheet Pile Model Pile
Web/flange width *a*	cm	0.2
Web/flange length *b*	cm	2.5
Pile cap width *c*	cm	2.5
Pile length *L*	cm	35.5
Elastic modulus of steel sheet pile *E*	GPa	210

**Table 4:** The technical parameters of OSI-S distributed optical fiber sensor.

Category	Index		Unit
Sensing length	50	100	m
Spatial resolution	1	10	mm
Sampling rate	4		Hz
Strain test accuracy	±1.0		με
Strain test range	±15,000		με
Temperature test accuracy	±0.1		°C
Temperature test range	−200~1200		°C

## Data Availability

Not applicable.
